# 

**Published:** 1896-09-05

**Authors:** 


					THE HOSPITAL. ABSOLUTELY PURE, therefore BEST, S*pt. 5th, 1896.
CADBURY'S *jr feM CADBURY'S
COCOA nP H, JT8 COCOA
" The standard of highest purity at present ? NO ALKALIES USED,
attainable."?Lancet.
!RWICH HOSPl TAL
No. 5197""* Vol. XX.
Saturday,
Sept. 5tii, 1896.
VklTH EXTRA KURSING SUPi LENIENT.
Per Anil am, 10b 6cL, post free.
[Registered at the General Post Offioe aa a Newspaper for MontUy Parts.ed.;
transmission in the United Kingdom ard Ahro.d.] Ditto per Annnm. 8s 6d.,poaiiree

				

